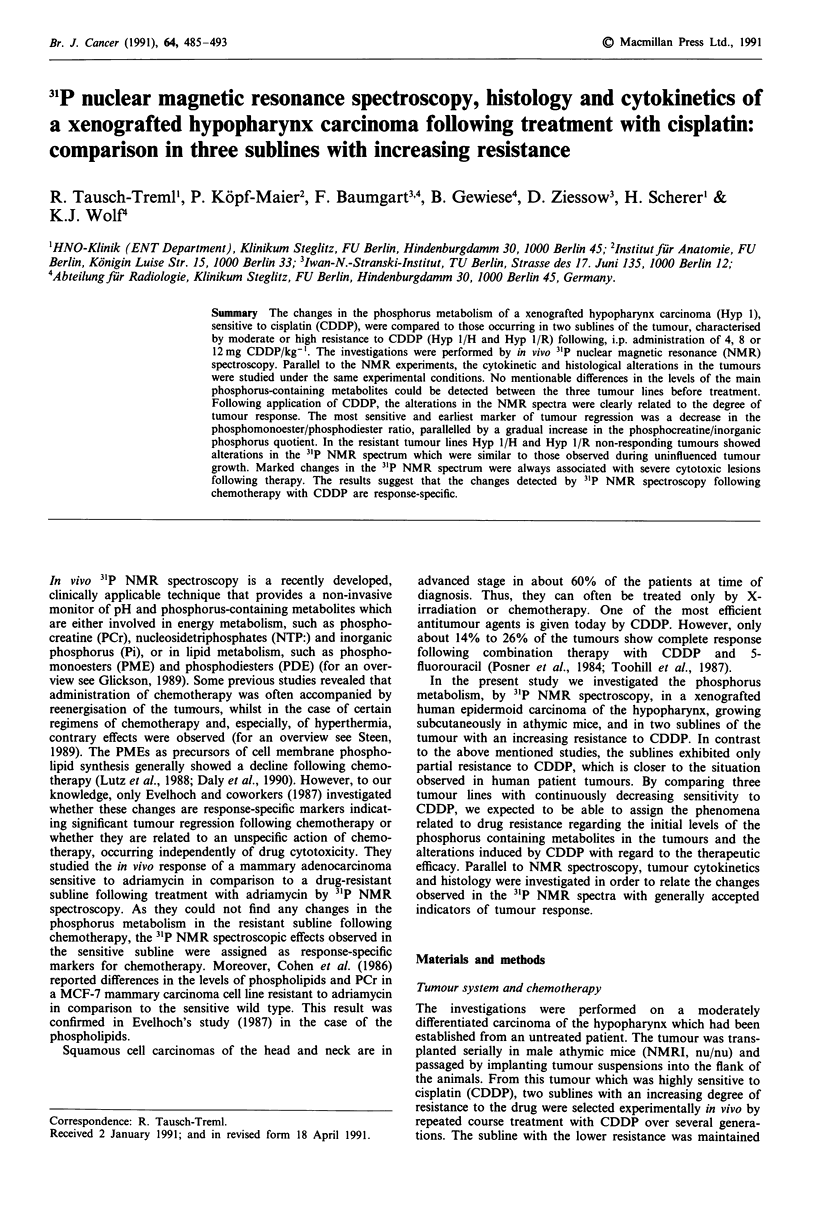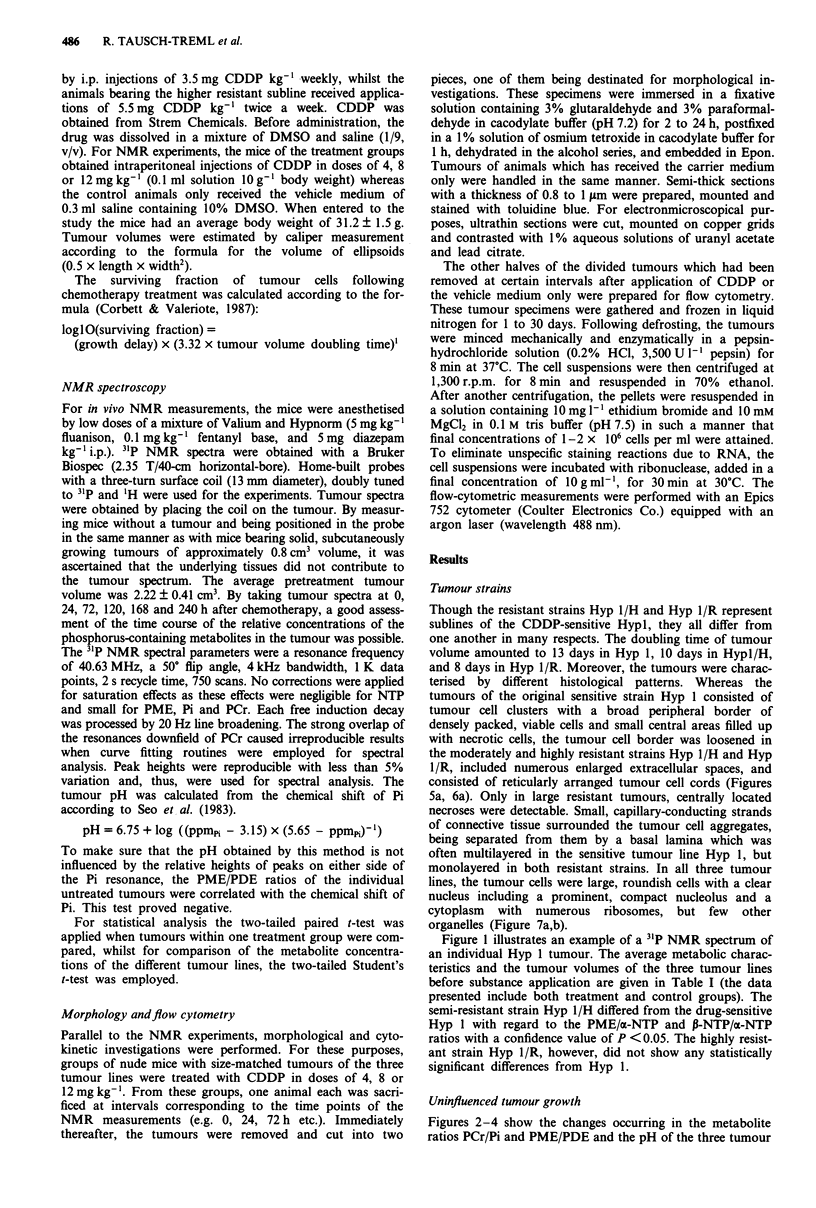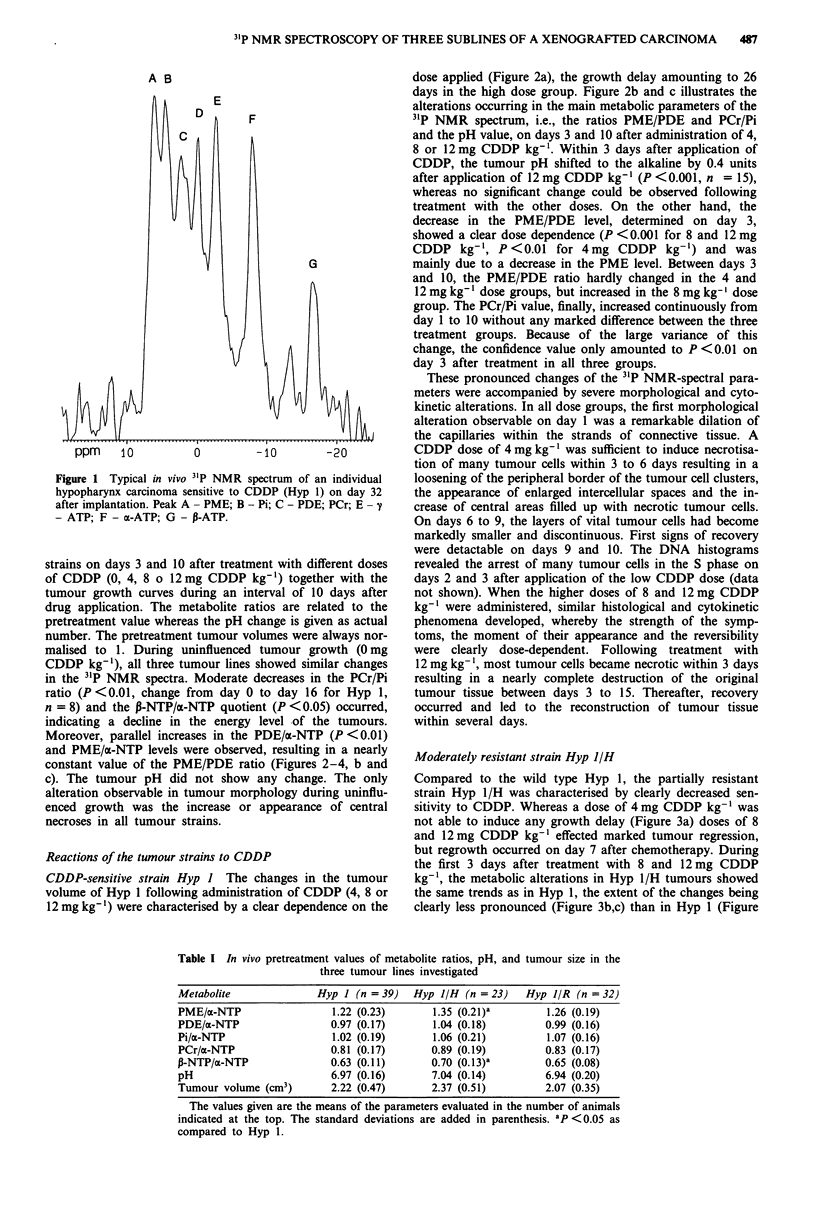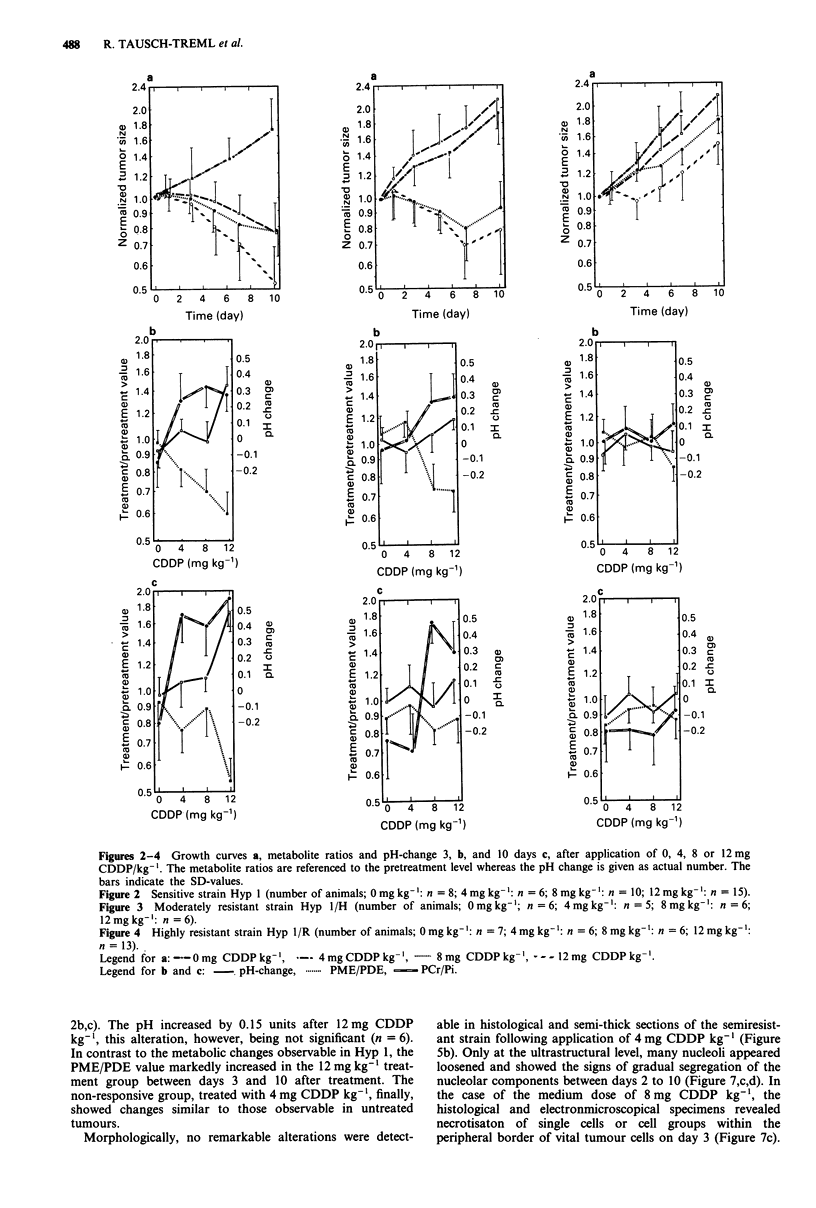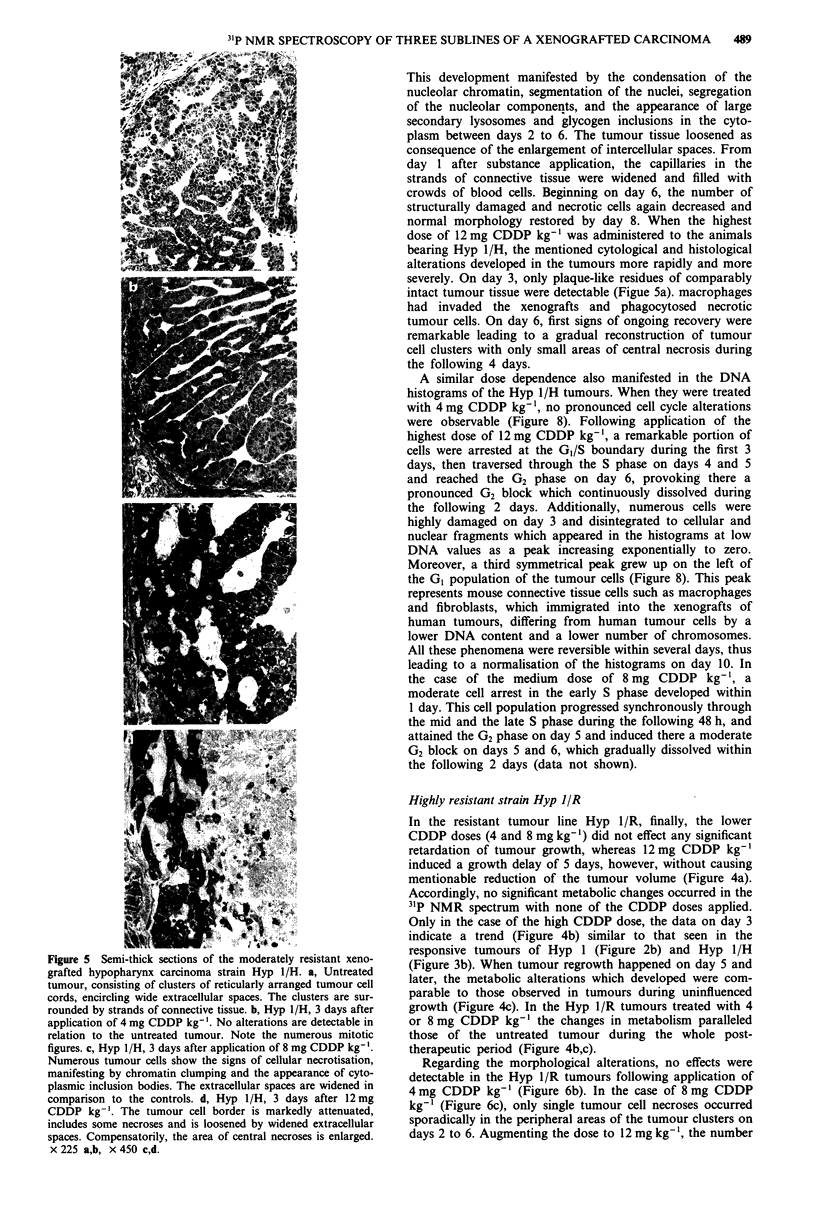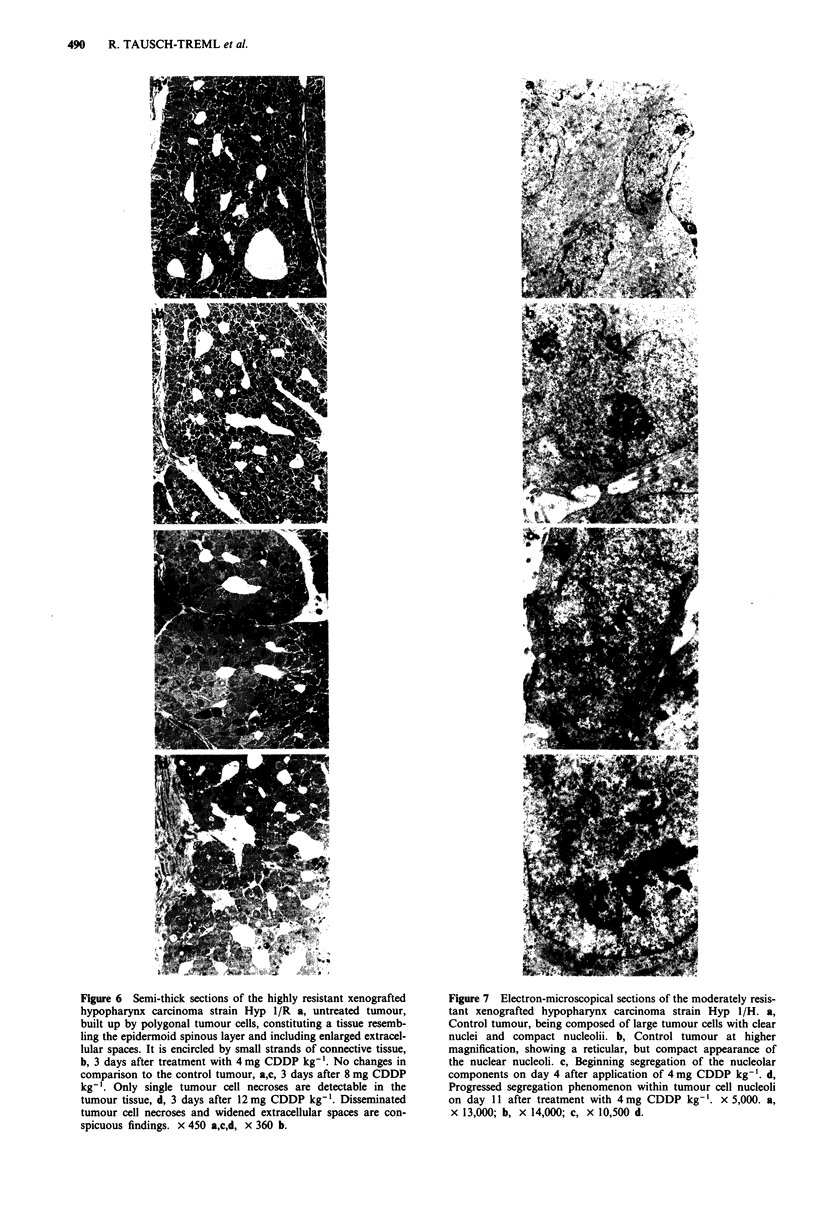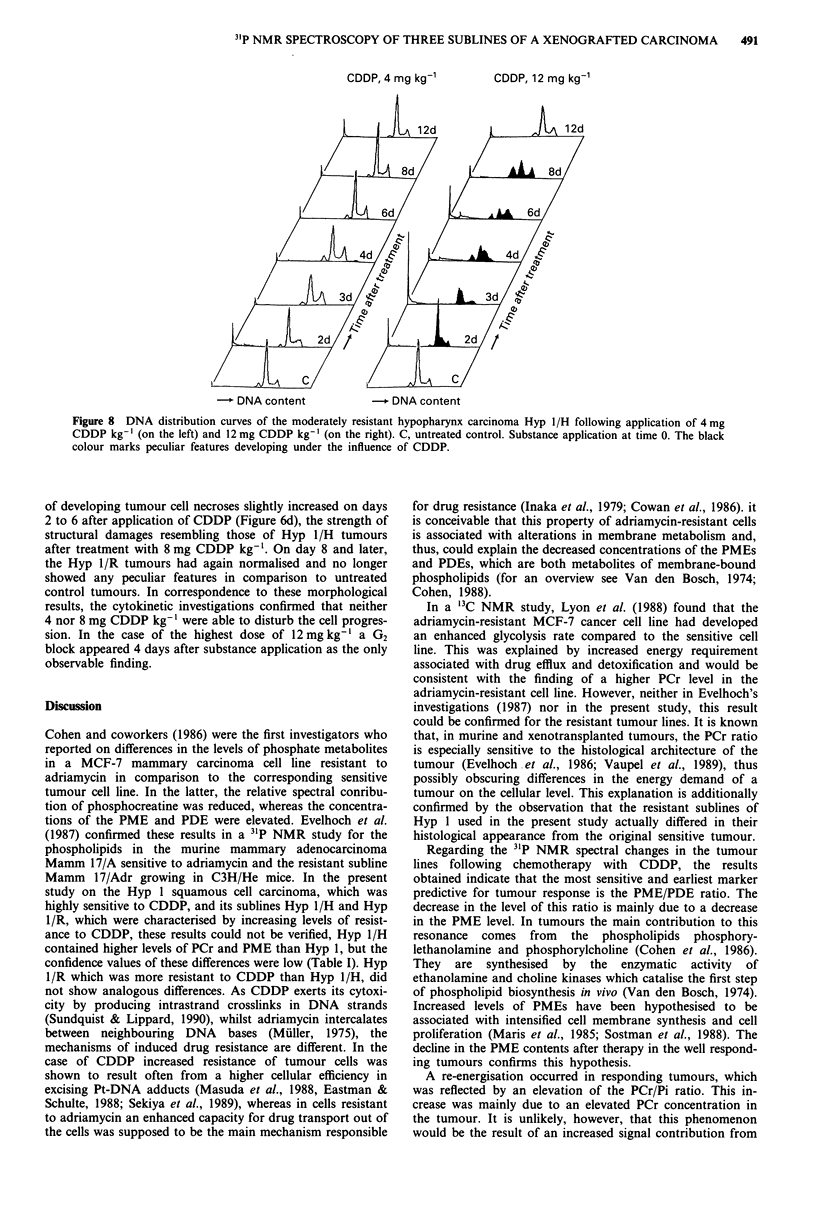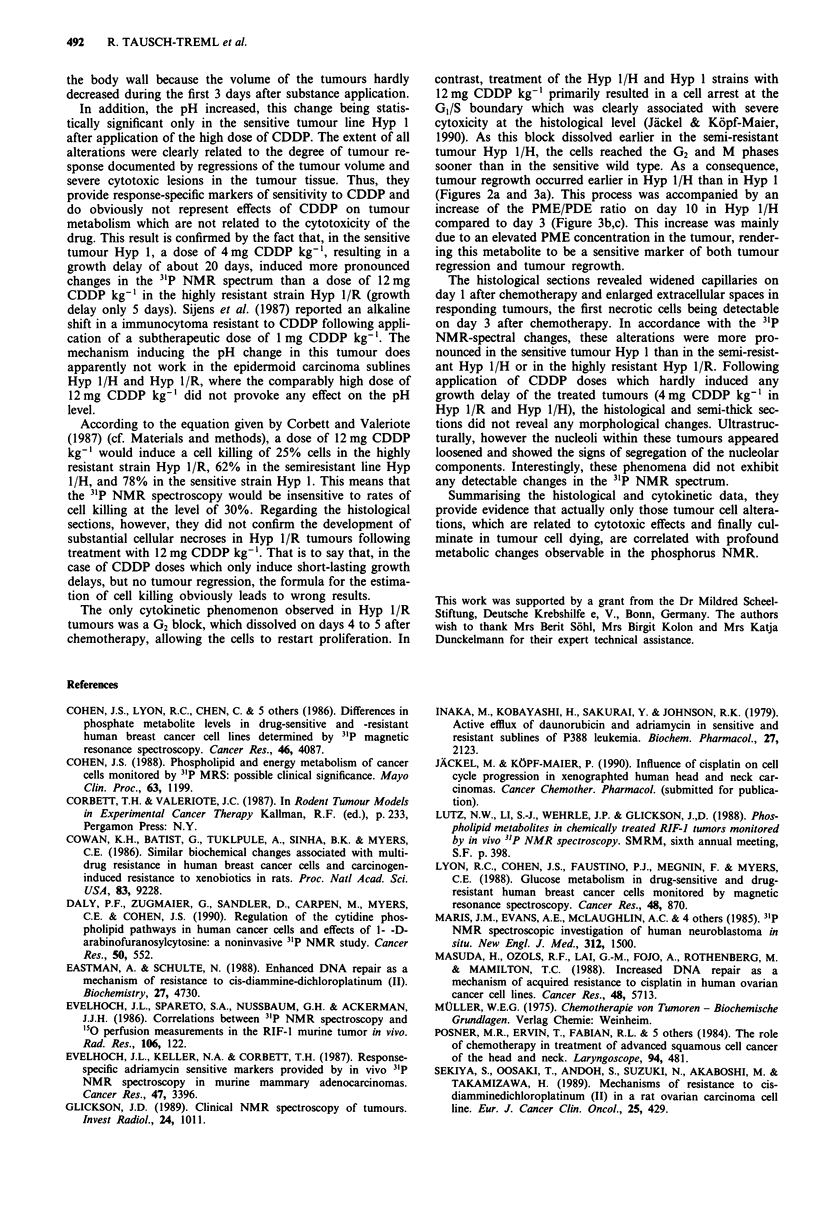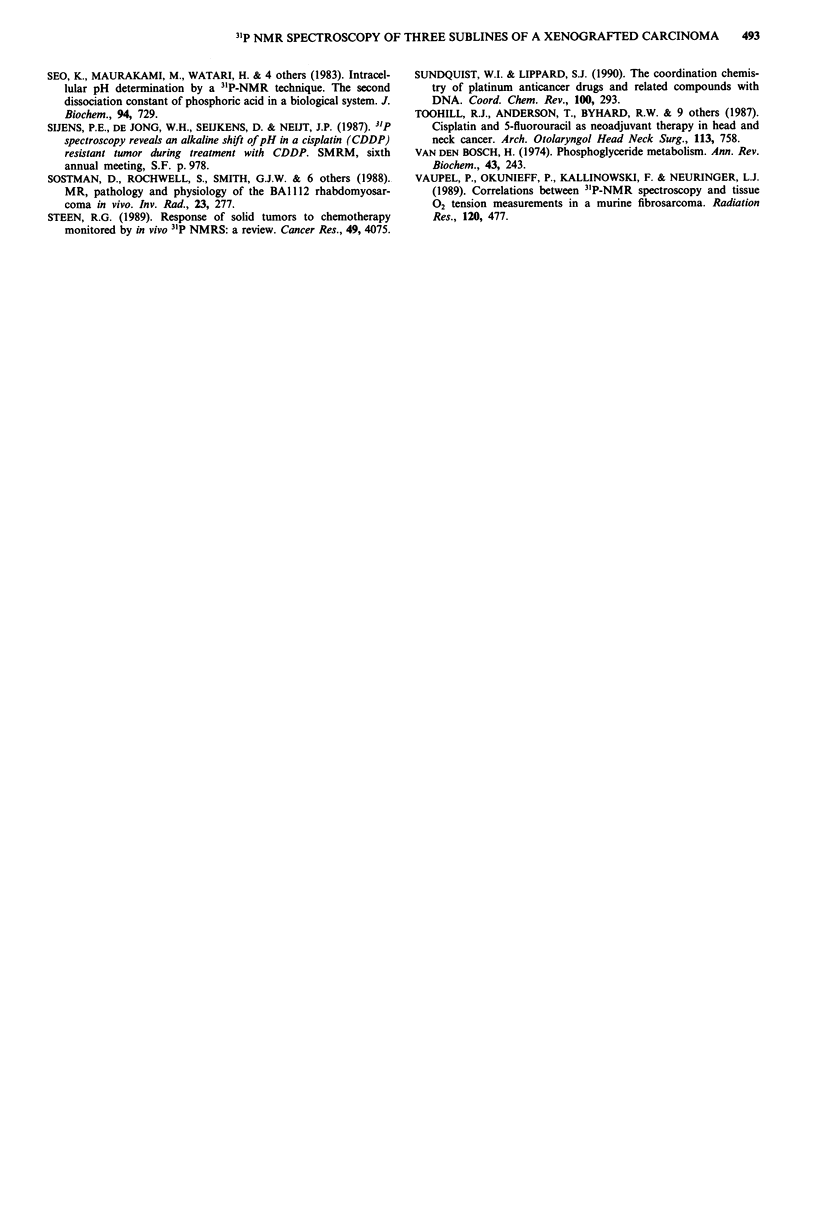# 31P nuclear magnetic resonance spectroscopy, histology and cytokinetics of a xenografted hypopharynx carcinoma following treatment with cisplatin: comparison in three sublines with increasing resistance.

**DOI:** 10.1038/bjc.1991.336

**Published:** 1991-09

**Authors:** R. Tausch-Treml, P. Köpf-Maier, F. Baumgart, B. Gewiese, D. Ziessow, H. Scherer, K. J. Wolf

**Affiliations:** HNO-Klinik ENT Department, FU Berlin, Germany.

## Abstract

**Images:**


Br. J. Cancer (1991), 64, 485-493 © Macmillan Press Ltd., 1991

### $^{31}$P nuclear magnetic resonance spectroscopy, histology and cytokinetics of a xenografted hypopharynx carcinoma following treatment with cisplatin: comparison in three sublines with increasing resistance

R. Tausch-Treml[1], P. Köpf-Maier[2], F. Baumgart[3,4], B. Gewiese[4], D. Ziessow[3], H. Scherer[1] & K.J. Wolf[4]

[1]*HNO-Klinik (ENT Department), Klinikum Steglitz, FU Berlin, Hindenburgdamm 30, 1000 Berlin 45;* [2]*Institut für Anatomie, FU Berlin, Königin Luise Str. 15, 1000 Berlin 33;* [3]*Iwan-N.-Stranski-Institut, TU Berlin, Strasse des 17. Juni 135, 1000 Berlin 12;* [4]*Abteilung für Radiologie, Klinikum Steglitz, FU Berlin, Hindenburgdamm 30, 1000 Berlin 45, Germany.*

**Summary** The changes in the phosphorus metabolism of a xenografted hypopharynx carcinoma (Hyp 1), sensitive to cisplatin (CDDP), were compared to those occurring in two sublines of the tumour, characterised by moderate or high resistance to CDDP (Hyp 1/H and Hyp 1/R) following, i.p. administration of 4, 8 or 12 mg CDDP/kg$^{-1}$. The investigations were performed by *in vivo* $^{31}$P nuclear magnetic resonance (NMR) spectroscopy. Parallel to the NMR experiments, the cytokinetic and histological alterations in the tumours were studied under the same experimental conditions. No mentionable differences in the levels of the main phosphorus-containing metabolites could be detected between the three tumour lines before treatment. Following application of CDDP, the alterations in the NMR spectra were clearly related to the degree of tumour response. The most sensitive and earliest marker of tumour regression was a decrease in the phosphomonoester/phosphodiester ratio, parallelled by a gradual increase in the phosphocreatine/inorganic phosphorus quotient. In the resistant tumour lines Hyp 1/H and Hyp 1/R non-responding tumours showed alterations in the $^{31}$P NMR spectrum which were similar to those observed during uninfluenced tumour growth. Marked changes in the $^{31}$P NMR spectrum were always associated with severe cytotoxic lesions following therapy. The results suggest that the changes detected by $^{31}$P NMR spectroscopy following chemotherapy with CDDP are response-specific.

*In vivo* $^{31}$P NMR spectroscopy is a recently developed, clinically applicable technique that provides a non-invasive monitor of pH and phosphorus-containing metabolites which are either involved in energy metabolism, such as phosphocreatine (PCr), nucleosidetriphosphates (NTP:) and inorganic phosphorus (Pi), or in lipid metabolism, such as phosphomonoesters (PME) and phosphodiesters (PDE) (for an overview see Glickson, 1989). Some previous studies revealed that administration of chemotherapy was often accompanied by reenergisation of the tumours, whilst in the case of certain regimens of chemotherapy and, especially, of hyperthermia, contrary effects were observed (for an overview see Steen, 1989). The PMEs as precursors of cell membrane phospholipid synthesis generally showed a decline following chemotherapy (Lutz *et al.*, 1988; Daly *et al.*, 1990). However, to our knowledge, only Evelhoch and coworkers (1987) investigated whether these changes are response-specific markers indicating significant tumour regression following chemotherapy or whether they are related to an unspecific action of chemotherapy, occurring independently of drug cytotoxicity. They studied the *in vivo* response of a mammary adenocarcinoma sensitive to adriamycin in comparison to a drug-resistant subline following treatment with adriamycin by $^{31}$P NMR spectroscopy. As they could not find any changes in the phosphorus metabolism in the resistant subline following chemotherapy, the $^{31}$P NMR spectroscopic effects observed in the sensitive subline were assigned as response-specific markers for chemotherapy. Moreover, Cohen *et al.* (1986) reported differences in the levels of phospholipids and PCr in a MCF-7 mammary carcinoma cell line resistant to adriamycin in comparison to the sensitive wild type. This result was confirmed in Evelhoch's study (1987) in the case of the phospholipids.

Squamous cell carcinomas of the head and neck are in advanced stage in about 60% of the patients at time of diagnosis. Thus, they can often be treated only by X-irradiation or chemotherapy. One of the most efficient antitumour agents is given today by CDDP. However, only about 14% to 26% of the tumours show complete response following combination therapy with CDDP and 5-fluorouracil (Posner *et al.*, 1984; Toohill *et al.*, 1987).

In the present study we investigated the phosphorus metabolism, by $^{31}$P NMR spectroscopy, in a xenografted human epidermoid carcinoma of the hypopharynx, growing subcutaneously in athymic mice, and in two sublines of the tumour with an increasing resistance to CDDP. In contrast to the above mentioned studies, the sublines exhibited only partial resistance to CDDP, which is closer to the situation observed in human patient tumours. By comparing three tumour lines with continuously decreasing sensitivity to CDDP, we expected to be able to assign the phenomena related to drug resistance regarding the initial levels of the phosphorus containing metabolites in the tumours and the alterations induced by CDDP with regard to the therapeutic efficacy. Parallel to NMR spectroscopy, tumour cytokinetics and histology were investigated in order to relate the changes observed in the $^{31}$P NMR spectra with generally accepted indicators of tumour response.

### Materials and methods

### *Tumour system and chemotherapy*

The investigations were performed on a moderately differentiated carcinoma of the hypopharynx which had been established from an untreated patient. The tumour was transplanted serially in male athymic mice (NMRI, nu/nu) and passaged by implanting tumour suspensions into the flank of the animals. From this tumour which was highly sensitive to cisplatin (CDDP), two sublines with an increasing degree of resistance to the drug were selected experimentally *in vivo* by repeated course treatment with CDDP over several generations. The subline with the lower resistance was maintained

Correspondence: R. Tausch-Treml.
Received 2 January 1991; and in revised form 18 April 1991.

by i.p. injections of 3.5 mg CDDP $kg^{-1}$ weekly, whilst the animals bearing the higher resistant subline received applications of 5.5 mg CDDP $kg^{-1}$ twice a week. CDDP was obtained from Strem Chemicals. Before administration, the drug was dissolved in a mixture of DMSO and saline (1/9, v/v). For NMR experiments, the mice of the treatment groups obtained intraperitoneal injections of CDDP in doses of 4, 8 or 12 mg $kg^{-1}$ (0.1 ml solution 10 $g^{-1}$ body weight) whereas the control animals only received the vehicle medium of 0.3 ml saline containing 10% DMSO. When entered to the study the mice had an average body weight of $31.2 \pm 1.5$ g. Tumour volumes were estimated by caliper measurement according to the formula for the volume of ellipsoids ($0.5 \times$ length $\times$ width$^2$).

The surviving fraction of tumour cells following chemotherapy treatment was calculated according to the formula (Corbett & Valeriote, 1987):

log1O(surviving fraction) =

(growth delay) $\times$ (3.32 $\times$ tumour volume doubling time)[1]

*NMR spectroscopy*

For *in vivo* NMR measurements, the mice were anesthetised by low doses of a mixture of Valium and Hypnorm (5 mg $kg^{-1}$ fluanison, 0.1 mg $kg^{-1}$ fentanyl base, and 5 mg diazepam $kg^{-1}$ i.p.). $^{31}$P NMR spectra were obtained with a Bruker Biospec (2.35 T/40-cm horizontal-bore). Home-built probes with a three-turn surface coil (13 mm diameter), doubly tuned to $^{31}$P and $^{1}$H were used for the experiments. Tumour spectra were obtained by placing the coil on the tumour. By measuring mice without a tumour and being positioned in the probe in the same manner as with mice bearing solid, subcutaneously growing tumours of approximately 0.8 $cm^3$ volume, it was ascertained that the underlying tissues did not contribute to the tumour spectrum. The average pretreatment tumour volume was $2.22 \pm 0.41$ $cm^3$. By taking tumour spectra at 0, 24, 72, 120, 168 and 240 h after chemotherapy, a good assessment of the time course of the relative concentrations of the phosphorus-containing metabolites in the tumour was possible. The $^{31}$P NMR spectral parameters were a resonance frequency of 40.63 MHz, a 50° flip angle, 4 kHz bandwidth, 1 K data points, 2 s recycle time, 750 scans. No corrections were applied for saturation effects as these effects were negligible for NTP and small for PME, Pi and PCr. Each free induction decay was processed by 20 Hz line broadening. The strong overlap of the resonances downfield of PCr caused irreproducible results when curve fitting routines were employed for spectral analysis. Peak heights were reproducible with less than 5% variation and, thus, were used for spectral analysis. The tumour pH was calculated from the chemical shift of Pi according to Seo *et al.* (1983).

$$pH = 6.75 + \log\left((ppm_{Pi} - 3.15) \times (5.65 - ppm_{Pi})^{-1}\right)$$

To make sure that the pH obtained by this method is not influenced by the relative heights of peaks on either side of the Pi resonance, the PME/PDE ratios of the individual untreated tumours were correlated with the chemical shift of Pi. This test proved negative.

For statistical analysis the two-tailed paired *t*-test was applied when tumours within one treatment group were compared, whilst for comparison of the metabolite concentrations of the different tumour lines, the two-tailed Student's *t*-test was employed.

*Morphology and flow cytometry*

Parallel to the NMR experiments, morphological and cytokinetic investigations were performed. For these purposes, groups of nude mice with size-matched tumours of the three tumour lines were treated with CDDP in doses of 4, 8 or 12 mg $kg^{-1}$. From these groups, one animal each was sacrificed at intervals corresponding to the time points of the NMR measurements (e.g. 0, 24, 72 h etc.). Immediately thereafter, the tumours were removed and cut into two pieces, one of them being destinated for morphological investigations. These specimens were immersed in a fixative solution containing 3% glutaraldehyde and 3% paraformaldehyde in cacodylate buffer (pH 7.2) for 2 to 24 h, postfixed in a 1% solution of osmium tetroxide in cacodylate buffer for 1 h, dehydrated in the alcohol series, and embedded in Epon. Tumours of animals which has received the carrier medium only were handled in the same manner. Semi-thick sections with a thickness of 0.8 to 1 μm were prepared, mounted and stained with toluidine blue. For electronmicroscopical purposes, ultrathin sections were cut, mounted on copper grids and contrasted with 1% aqueous solutions of uranyl acetate and lead citrate.

The other halves of the divided tumours which had been removed at certain intervals after application of CDDP or the vehicle medium only were prepared for flow cytometry. These tumour specimens were gathered and frozen in liquid nitrogen for 1 to 30 days. Following defrosting, the tumours were minced mechanically and enzymatically in a pepsin-hydrochloride solution (0.2% HCl, 3,500 U $l^{-1}$ pepsin) for 8 min at 37°C. The cell suspensions were then centrifuged at 1,300 r.p.m. for 8 min and resuspended in 70% ethanol. After another centrifugation, the pellets were resuspended in a solution containing 10 mg $l^{-1}$ ethidium bromide and 10 mM $MgCl_2$ in 0.1 M tris buffer (pH 7.5) in such a manner that final concentrations of $1-2 \times 10^6$ cells per ml were attained. To eliminate unspecific staining reactions due to RNA, the cell suspensions were incubated with ribonuclease, added in a final concentration of 10 g $ml^{-1}$, for 30 min at 30°C. The flow-cytometric measurements were performed with an Epics 752 cytometer (Coulter Electronics Co.) equipped with an argon laser (wavelength 488 nm).

### Results

*Tumour strains*

Though the resistant strains Hyp 1/H and Hyp 1/R represent sublines of the CDDP-sensitive Hyp1, they all differ from one another in many respects. The doubling time of tumour volume amounted to 13 days in Hyp 1, 10 days in Hyp1/H, and 8 days in Hyp 1/R. Moreover, the tumours were characterised by different histological patterns. Whereas the tumours of the original sensitive strain Hyp 1 consisted of tumour cell clusters with a broad peripheral border of densely packed, viable cells and small central areas filled up with necrotic cells, the tumour cell border was loosened in the moderately and highly resistant strains Hyp 1/H and Hyp 1/R, included numerous enlarged extracellular spaces, and consisted of reticularly arranged tumour cell cords (Figures 5a, 6a). Only in large resistant tumours, centrally located necroses were detectable. Small, capillary-conducting strands of connective tissue surrounded the tumour cell aggregates, being separated from them by a basal lamina which was often multilayered in the sensitive tumour line Hyp 1, but monolayered in both resistant strains. In all three tumour lines, the tumour cells were large, roundish cells with a clear nucleus including a prominent, compact nucleolus and a cytoplasm with numerous ribosomes, but few other organelles (Figure 7a,b).

Figure 1 illustrates an example of a $^{31}$P NMR spectrum of an individual Hyp 1 tumour. The average metabolic characteristics and the tumour volumes of the three tumour lines before substance application are given in Table I (the data presented include both treatment and control groups). The semi-resistant strain Hyp 1/H differed from the drug-sensitive Hyp 1 with regard to the PME/α-NTP and β-NTP/α-NTP ratios with a confidence value of $P < 0.05$. The highly resistant strain Hyp 1/R, however, did not show any statistically significant differences from Hyp 1.

*Uninfluenced tumour growth*

Figures 2–4 show the changes occurring in the metabolite ratios PCr/Pi and PME/PDE and the pH of the three tumour

**Figure 1** Typical *in vivo* $^{31}$P NMR spectrum of an individual hypopharynx carcinoma sensitive to CDDP (Hyp 1) on day 32 after implantation. Peak A – PME; B – Pi; C – PDE; PCr; E – $\gamma$ – ATP; F – $\alpha$-ATP; G – $\beta$-ATP.

strains on days 3 and 10 after treatment with different doses of CDDP (0, 4, 8 o 12 mg CDDP $kg^{-1}$) together with the tumour growth curves during an interval of 10 days after drug application. The metabolite ratios are related to the pretreatment value whereas the pH change is given as actual number. The pretreatment tumour volumes were always normalised to 1. During uninfluenced tumour growth (0 mg CDDP $kg^{-1}$), all three tumour lines showed similar changes in the $^{31}$P NMR spectra. Moderate decreases in the PCr/Pi ratio ($P < 0.01$, change from day 0 to day 16 for Hyp 1, $n = 8$) and the $\beta$-NTP/$\alpha$-NTP quotient ($P < 0.05$) occurred, indicating a decline in the energy level of the tumours. Moreover, parallel increases in the PDE/$\alpha$-NTP ($P < 0.01$) and PME/$\alpha$-NTP levels were observed, resulting in a nearly constant value of the PME/PDE ratio (Figures 2–4, b and c). The tumour pH did not show any change. The only alteration observable in tumour morphology during uninfluenced growth was the increase or appearance of central necroses in all tumour strains.

*Reactions of the tumour strains to CDDP*

*CDDP-sensitive strain Hyp 1* The changes in the tumour volume of Hyp 1 following administration of CDDP (4, 8 or 12 mg $kg^{-1}$) were characterised by a clear dependence on the dose applied (Figure 2a), the growth delay amounting to 26 days in the high dose group. Figure 2b and c illustrates the alterations occurring in the main metabolic parameters of the $^{31}$P NMR spectrum, i.e., the ratios PME/PDE and PCr/Pi and the pH value, on days 3 and 10 after administration of 4, 8 or 12 mg CDDP $kg^{-1}$. Within 3 days after application of CDDP, the tumour pH shifted to the alkaline by 0.4 units after application of 12 mg CDDP $kg^{-1}$ ($P < 0.001$, $n = 15$), whereas no significant change could be observed following treatment with the other doses. On the other hand, the decrease in the PME/PDE level, determined on day 3, showed a clear dose dependence ($P < 0.001$ for 8 and 12 mg CDDP $kg^{-1}$, $P < 0.01$ for 4 mg CDDP $kg^{-1}$) and was mainly due to a decrease in the PME level. Between days 3 and 10, the PME/PDE ratio hardly changed in the 4 and 12 mg $kg^{-1}$ dose groups, but increased in the 8 mg $kg^{-1}$ dose group. The PCr/Pi value, finally, increased continuously from day 1 to 10 without any marked difference between the three treatment groups. Because of the large variance of this change, the confidence value only amounted to $P < 0.01$ on day 3 after treatment in all three groups.

These pronounced changes of the $^{31}$P NMR-spectral parameters were accompanied by severe morphological and cytokinetic alterations. In all dose groups, the first morphological alteration observable on day 1 was a remarkable dilation of the capillaries within the strands of connective tissue. A CDDP dose of 4 mg $kg^{-1}$ was sufficient to induce necrotisation of many tumour cells within 3 to 6 days resulting in a loosening of the peripheral border of the tumour cell clusters, the appearance of enlarged intercellular spaces and the increase of central areas filled up with necrotic tumour cells. On days 6 to 9, the layers of vital tumour cells had become markedly smaller and discontinuous. First signs of recovery were detactable on days 9 and 10. The DNA histograms revealed the arrest of many tumour cells in the S phase on days 2 and 3 after application of the low CDDP dose (data not shown). When the higher doses of 8 and 12 mg CDDP $kg^{-1}$ were administered, similar histological and cytokinetic phenomena developed, whereby the strength of the symptoms, the moment of their appearance and the reversibility were clearly dose-dependent. Following treatment with 12 mg $kg^{-1}$, most tumour cells became necrotic within 3 days resulting in a nearly complete destruction of the original tumour tissue between days 3 to 15. Thereafter, recovery occurred and led to the reconstruction of tumour tissue within several days.

*Moderately resistant strain Hyp 1/H*

Compared to the wild type Hyp 1, the partially resistant strain Hyp 1/H was characterised by clearly decreased sensitivity to CDDP. Whereas a dose of 4 mg CDDP $kg^{-1}$ was not able to induce any growth delay (Figure 3a) doses of 8 and 12 mg CDDP $kg^{-1}$ effected marked tumour regression, but regrowth occurred on day 7 after chemotherapy. During the first 3 days after treatment with 8 and 12 mg CDDP $kg^{-1}$, the metabolic alterations in Hyp 1/H tumours showed the same trends as in Hyp 1, the extent of the changes being clearly less pronounced (Figure 3b,c) than in Hyp 1 (Figure

**Table I** *In vivo* pretreatment values of metabolite ratios, pH, and tumour size in the three tumour lines investigated

| *Metabolite* | *Hyp 1 (n = 39)* | *Hyp 1/H (n = 23)* | *Hyp 1/R (n = 32)* |
|---|---|---|---|
| PME/$\alpha$-NTP | 1.22 (0.23) | 1.35 (0.21)[a] | 1.26 (0.19) |
| PDE/$\alpha$-NTP | 0.97 (0.17) | 1.04 (0.18) | 0.99 (0.16) |
| Pi/$\alpha$-NTP | 1.02 (0.19) | 1.06 (0.21) | 1.07 (0.16) |
| PCr/$\alpha$-NTP | 0.81 (0.17) | 0.89 (0.19) | 0.83 (0.17) |
| $\beta$-NTP/$\alpha$-NTP | 0.63 (0.11) | 0.70 (0.13)[a] | 0.65 (0.08) |
| pH | 6.97 (0.16) | 7.04 (0.14) | 6.94 (0.20) |
| Tumour volume ($cm^3$) | 2.22 (0.47) | 2.37 (0.51) | 2.07 (0.35) |

The values given are the means of the parameters evaluated in the number of animals indicated at the top. The standard deviations are added in parenthesis. [a]$P < 0.05$ as compared to Hyp 1.

**Figures 2–4** Growth curves **a**, metabolite ratios and pH-change 3, **b**, and 10 days **c**, after application of 0, 4, 8 or 12 mg CDDP/kg$^{-1}$. The metabolite ratios are referenced to the pretreatment level whereas the pH change is given as actual number. The bars indicate the SD-values.

**Figure 2** Sensitive strain Hyp 1 (number of animals; 0 mg kg$^{-1}$: $n = 8$; 4 mg kg$^{-1}$: $n = 6$; 8 mg kg$^{-1}$: $n = 10$; 12 mg kg$^{-1}$: $n = 15$).

**Figure 3** Moderately resistant strain Hyp 1/H (number of animals; 0 mg kg$^{-1}$; $n = 6$; 4 mg kg$^{-1}$: $n = 5$; 8 mg kg$^{-1}$: $n = 6$; 12 mg kg$^{-1}$: $n = 6$).

**Figure 4** Highly resistant strain Hyp 1/R (number of animals; 0 mg kg$^{-1}$: $n = 7$; 4 mg kg$^{-1}$: $n = 6$; 8 mg kg$^{-1}$: $n = 6$; 12 mg kg$^{-1}$: $n = 13$).

Legend for **a**: —·— 0 mg CDDP kg$^{-1}$, ·— 4 mg CDDP kg$^{-1}$, ······· 8 mg CDDP kg$^{-1}$, - - - 12 mg CDDP kg$^{-1}$.

Legend for **b** and **c**: ——. pH-change, ······· PME/PDE, ═══ PCr/Pi.

2b,c). The pH increased by 0.15 units after 12 mg CDDP kg$^{-1}$, this alteration, however, being not significant ($n = 6$). In contrast to the metabolic changes observable in Hyp 1, the PME/PDE value markedly increased in the 12 mg kg$^{-1}$ treatment group between days 3 and 10 after treatment. The non-responsive group, treated with 4 mg CDDP kg$^{-1}$, finally, showed changes similar to those observable in untreated tumours.

Morphologically, no remarkable alterations were detectable in histological and semi-thick sections of the semiresistant strain following application of 4 mg CDDP kg$^{-1}$ (Figure 5b). Only at the ultrastructural level, many nucleoli appeared loosened and showed the signs of gradual segregation of the nucleolar components between days 2 to 10 (Figure 7,c,d). In the case of the medium dose of 8 mg CDDP kg$^{-1}$, the histological and electronmicroscopical specimens revealed necrotisaton of single cells or cell groups within the peripheral border of vital tumour cells on day 3 (Figure 7c).

**Figure 5** Semi-thick sections of the moderately resistant xenografted hypopharynx carcinoma strain Hyp 1/H. **a**, Untreated tumour, consisting of clusters of reticularly arranged tumour cell cords, encircling wide extracellular spaces. The clusters are surrounded by strands of connective tissue. **b**, Hyp 1/H, 3 days after application of 4 mg CDDP $kg^{-1}$. No alterations are detectable in relation to the untreated tumour. Note the numerous mitotic figures. **c**, Hyp 1/H, 3 days after application of 8 mg CDDP $kg^{-1}$. Numerous tumour cells show the signs of cellular necrotisation, manifesting by chromatin clumping and the appearance of cytoplasmic inclusion bodies. The extracellular spaces are widened in comparison to the controls. **d**, Hyp 1/H, 3 days after 12 mg CDDP $kg^{-1}$. The tumour cell border is markedly attenuated, includes some necroses and is loosened by widened extracellular spaces. Compensatorily, the area of central necroses is enlarged. × 225 **a**,**b**, × 450 **c**,**d**.

This development manifested by the condensation of the nucleolar chromatin, segmentation of the nuclei, segregation of the nucleolar components, and the appearance of large secondary lysosomes and glycogen inclusions in the cytoplasm between days 2 to 6. The tumour tissue loosened as consequence of the enlargement of intercellular spaces. From day 1 after substance application, the capillaries in the strands of connective tissue were widened and filled with crowds of blood cells. Beginning on day 6, the number of structurally damaged and necrotic cells again decreased and normal morphology restored by day 8. When the highest dose of 12 mg CDDP $kg^{-1}$ was administered to the animals bearing Hyp 1/H, the mentioned cytological and histological alterations developed in the tumours more rapidly and more severely. On day 3, only plaque-like residues of comparably intact tumour tissue were detectable (Figue 5a). macrophages had invaded the xenografts and phagocytosed necrotic tumour cells. On day 6, first signs of ongoing recovery were remarkable leading to a gradual reconstruction of tumour cell clusters with only small areas of central necrosis during the following 4 days.

A similar dose dependence also manifested in the DNA histograms of the Hyp 1/H tumours. When they were treated with 4 mg CDDP $kg^{-1}$, no pronounced cell cycle alterations were observable (Figure 8). Following application of the highest dose of 12 mg CDDP $kg^{-1}$, a remarkable portion of cells were arrested at the $G_1$/S boundary during the first 3 days, then traversed through the S phase on days 4 and 5 and reached the $G_2$ phase on day 6, provoking there a pronounced $G_2$ block which continuously dissolved during the following 2 days. Additionally, numerous cells were highly damaged on day 3 and disintegrated to cellular and nuclear fragments which appeared in the histograms at low DNA values as a peak increasing exponentially to zero. Moreover, a third symmetrical peak grew up on the left of the $G_1$ population of the tumour cells (Figure 8). This peak represents mouse connective tissue cells such as macrophages and fibroblasts, which immigrated into the xenografts of human tumours, differing from human tumour cells by a lower DNA content and a lower number of chromosomes. All these phenomena were reversible within several days, thus leading to a normalisation of the histograms on day 10. In the case of the medium dose of 8 mg CDDP $kg^{-1}$, a moderate cell arrest in the early S phase developed within 1 day. This cell population progressed synchronously through the mid and the late S phase during the following 48 h, and attained the $G_2$ phase on day 5 and induced there a moderate $G_2$ block on days 5 and 6, which gradually dissolved within the following 2 days (data not shown).

### *Highly resistant strain Hyp 1/R*

In the resistant tumour line Hyp 1/R, finally, the lower CDDP doses (4 and 8 mg $kg^{-1}$) did not effect any significant retardation of tumour growth, whereas 12 mg CDDP $kg^{-1}$ induced a growth delay of 5 days, however, without causing mentionable reduction of the tumour volume (Figure 4a). Accordingly, no significant metabolic changes occurred in the $^{31}$P NMR spectrum with none of the CDDP doses applied. Only in the case of the high CDDP dose, the data on day 3 indicate a trend (Figure 4b) similar to that seen in the responsive tumours of Hyp 1 (Figure 2b) and Hyp 1/H (Figure 3b). When tumour regrowth happened on day 5 and later, the metabolic alterations which developed were comparable to those observed in tumours during uninfluenced growth (Figure 4c). In the Hyp 1/R tumours treated with 4 or 8 mg CDDP $kg^{-1}$ the changes in metabolism paralleled those of the untreated tumour during the whole post-therapeutic period (Figure 4b,c).

Regarding the morphological alterations, no effects were detectable in the Hyp 1/R tumours following application of 4 mg CDDP $kg^{-1}$ (Figure 6b). In the case of 8 mg CDDP $kg^{-1}$ (Figure 6c), only single tumour cell necroses occurred sporadically in the peripheral areas of the tumour clusters on days 2 to 6. Augmenting the dose to 12 mg $kg^{-1}$, the number

**Figure 6** Semi-thick sections of the highly resistant xenografted hypopharynx carcinoma strain Hyp 1/R **a**, untreated tumour, built up by polygonal tumour cells, constituting a tissue resembling the epidermoid spinous layer and including enlarged extracellular spaces. It is encircled by small strands of connective tissue, **b**, 3 days after treatment with 4 mg CDDP $kg^{-1}$. No changes in comparison to the control tumour, **a**,**c**, 3 days after 8 mg CDDP $kg^{-1}$. Only single tumour cell necroses are detectable in the tumour tissue, **d**, 3 days after 12 mg CDDP $kg^{-1}$. Disseminated tumour cell necroses and widened extracellular spaces are conspicuous findings. × 450 **a**,**c**,**d**, × 360 **b**.

**Figure 7** Electron-microscopical sections of the moderately resistant xenografted hypopharynx carcinoma strain Hyp 1/H. **a**, Control tumour, being composed of large tumour cells with clear nuclei and compact nucleolii. **b**, Control tumour at higher magnification, showing a reticular, but compact appearance of the nuclear nucleoli. **c**, Beginning segregation of the nucleolar components on day 4 after application of 4 mg CDDP $kg^{-1}$. **d**, Progressed segregation phenomenon within tumour cell nucleoli on day 11 after treatment with 4 mg CDDP $kg^{-1}$. × 5,000. **a**, × 13,000; **b**, × 14,000; **c**, × 10,500 **d**.

**Figure 8** DNA distribution curves of the moderately resistant hypopharynx carcinoma Hyp 1/H following application of 4 mg CDDP kg$^{-1}$ (on the left) and 12 mg CDDP kg$^{-1}$ (on the right). C, untreated control. Substance application at time 0. The black colour marks peculiar features developing under the influence of CDDP.

of developing tumour cell necroses slightly increased on days 2 to 6 after application of CDDP (Figure 6d), the strength of structural damages resembling those of Hyp 1/H tumours after treatment with 8 mg CDDP kg$^{-1}$. On day 8 and later, the Hyp 1/R tumours had again normalised and no longer showed any peculiar features in comparison to untreated control tumours. In correspondence to these morphological results, the cytokinetic investigations confirmed that neither 4 nor 8 mg CDDP kg$^{-1}$ were able to disturb the cell progression. In the case of the highest dose of 12 mg kg$^{-1}$ a $G_2$ block appeared 4 days after substance application as the only observable finding.

### Discussion

Cohen and coworkers (1986) were the first investigators who reported on differences in the levels of phosphate metabolites in a MCF-7 mammary carcinoma cell line resistant to adriamycin in comparison to the corresponding sensitive tumour cell line. In the latter, the relative spectral conribution of phosphocreatine was reduced, whereas the concentrations of the PME and PDE were elevated. Evelhoch *et al.* (1987) confirmed these results in a $^{31}$P NMR study for the phospholipids in the murine mammary adenocarcinoma Mamm 17/A sensitive to adriamycin and the resistant subline Mamm 17/Adr growing in C3H/He mice. In the present study on the Hyp 1 squamous cell carcinoma, which was highly sensitive to CDDP, and its sublines Hyp 1/H and Hyp 1/R, which were characterised by increasing levels of resistance to CDDP, these results could not be verified, Hyp 1/H contained higher levels of PCr and PME than Hyp 1, but the confidence values of these differences were low (Table I). Hyp 1/R which was more resistant to CDDP than Hyp 1/H, did not show analogous differences. As CDDP exerts its cytoxicity by producing intrastrand crosslinks in DNA strands (Sundquist & Lippard, 1990), whilst adriamycin intercalates between neighbouring DNA bases (Müller, 1975), the mechanisms of induced drug resistance are different. In the case of CDDP increased resistance of tumour cells was shown to result often from a higher cellular efficiency in excising Pt-DNA adducts (Masuda *et al.*, 1988, Eastman & Schulte, 1988; Sekiya *et al.*, 1989), whereas in cells resistant to adriamycin an enhanced capacity for drug transport out of the cells was supposed to be the main mechanism responsible for drug resistance (Inaka *et al.*, 1979; Cowan *et al.*, 1986). it is conceivable that this property of adriamycin-resistant cells is associated with alterations in membrane metabolism and, thus, could explain the decreased concentrations of the PMEs and PDEs, which are both metabolites of membrane-bound phospholipids (for an overview see Van den Bosch, 1974; Cohen, 1988).

In a $^{13}$C NMR study, Lyon *et al.* (1988) found that the adriamycin-resistant MCF-7 cancer cell line had developed an enhanced glycolysis rate compared to the sensitive cell line. This was explained by increased energy requirement associated with drug efflux and detoxification and would be consistent with the finding of a higher PCr level in the adriamycin-resistant cell line. However, neither in Evelhoch's investigations (1987) nor in the present study, this result could be confirmed for the resistant tumour lines. It is known that, in murine and xenotransplanted tumours, the PCr ratio is especially sensitive to the histological architecture of the tumour (Evelhoch *et al.*, 1986; Vaupel *et al.*, 1989), thus possibly obscuring differences in the energy demand of a tumour on the cellular level. This explanation is additionally confirmed by the observation that the resistant sublines of Hyp 1 used in the present study actually differed in their histological appearance from the original sensitive tumour.

Regarding the $^{31}$P NMR spectral changes in the tumour lines following chemotherapy with CDDP, the results obtained indicate that the most sensitive and earliest marker predictive for tumour response is the PME/PDE ratio. The decrease in the level of this ratio is mainly due to a decrease in the PME level. In tumours the main contribution to this resonance comes from the phospholipids phosphorylethanolamine and phosphorylcholine (Cohen *et al.*, 1986). They are synthesised by the enzymatic activity of ethanolamine and choline kinases which catalise the first step of phospholipid biosynthesis *in vivo* (Van den Bosch, 1974). Increased levels of PMEs have been hypothesised to be associated with intensified cell membrane synthesis and cell proliferation (Maris *et al.*, 1985; Sostman *et al.*, 1988). The decline in the PME contents after therapy in the well responding tumours confirms this hypothesis.

A re-energisation occurred in responding tumours, which was reflected by an elevation of the PCr/Pi ratio. This increase was mainly due to an elevated PCr concentration in the tumour. It is unlikely, however, that this phenomenon would be the result of an increased signal contribution from the body wall because the volume of the tumours hardly decreased during the first 3 days after substance application.

In addition, the pH increased, this change being statistically significant only in the sensitive tumour line Hyp 1 after application of the high dose of CDDP. The extent of all alterations were clearly related to the degree of tumour response documented by regressions of the tumour volume and severe cytotoxic lesions in the tumour tissue. Thus, they provide response-specific markers of sensitivity to CDDP and do obviously not represent effects of CDDP on tumour metabolism which are not related to the cytotoxicity of the drug. This result is confirmed by the fact that, in the sensitive tumour Hyp 1, a dose of 4 mg CDDP $kg^{-1}$, resulting in a growth delay of about 20 days, induced more pronounced changes in the $^{31}P$ NMR spectrum than a dose of 12 mg CDDP $kg^{-1}$ in the highly resistant strain Hyp 1/R (growth delay only 5 days). Sijens *et al.* (1987) reported an alkaline shift in a immunocytoma resistant to CDDP following application of a subtherapeutic dose of 1 mg CDDP $kg^{-1}$. The mechanism inducing the pH change in this tumour does apparently not work in the epidermoid carcinoma sublines Hyp 1/H and Hyp 1/R, where the comparably high dose of 12 mg CDDP $kg^{-1}$ did not provoke any effect on the pH level.

According to the equation given by Corbett and Valeriote (1987) (cf. Materials and methods), a dose of 12 mg CDDP $kg^{-1}$ would induce a cell killing of 25% cells in the highly resistant strain Hyp 1/R, 62% in the semiresistant line Hyp 1/H, and 78% in the sensitive strain Hyp 1. This means that the $^{31}P$ NMR spectroscopy would be insensitive to rates of cell killing at the level of 30%. Regarding the histological sections, however, they did not confirm the development of substantial cellular necroses in Hyp 1/R tumours following treatment with 12 mg CDDP $kg^{-1}$. That is to say that, in the case of CDDP doses which only induce short-lasting growth delays, but no tumour regression, the formula for the estimation of cell killing obviously leads to wrong results.

The only cytokinetic phenomenon observed in Hyp 1/R tumours was a $G_2$ block, which dissolved on days 4 to 5 after chemotherapy, allowing the cells to restart proliferation. In contrast, treatment of the Hyp 1/H and Hyp 1 strains with 12 mg CDDP $kg^{-1}$ primarily resulted in a cell arrest at the $G_1$/S boundary which was clearly associated with severe cytoxicity at the histological level (Jäckel & Köpf-Maier, 1990). As this block dissolved earlier in the semi-resistant tumour Hyp 1/H, the cells reached the $G_2$ and M phases sooner than in the sensitive wild type. As a consequence, tumour regrowth occurred earlier in Hyp 1/H than in Hyp 1 (Figures 2a and 3a). This process was accompanied by an increase of the PME/PDE ratio on day 10 in Hyp 1/H compared to day 3 (Figure 3b,c). This increase was mainly due to an elevated PME concentration in the tumour, rendering this metabolite to be a sensitive marker of both tumour regression and tumour regrowth.

The histological sections revealed widened capillaries on day 1 after chemotherapy and enlarged extracellular spaces in responding tumours, the first necrotic cells being detectable on day 3 after chemotherapy. In accordance with the $^{31}P$ NMR-spectral changes, these alterations were more pronounced in the sensitive tumour Hyp 1 than in the semi-resistant Hyp 1/H or in the highly resistant Hyp 1/R. Following application of CDDP doses which hardly induced any growth delay of the treated tumours (4 mg CDDP $kg^{-1}$ in Hyp 1/R and Hyp 1/H), the histological and semi-thick sections did not reveal any morphological changes. Ultrastructurally, however the nucleoli within these tumours appeared loosened and showed the signs of segregation of the nucleolar components. Interestingly, these phenomena did not exhibit any detectable changes in the $^{31}P$ NMR spectrum.

Summarising the histological and cytokinetic data, they provide evidence that actually only those tumour cell alterations, which are related to cytotoxic effects and finally culminate in tumour cell dying, are correlated with profound metabolic changes observable in the phosphorus NMR.

This work was supported by a grant from the Dr Mildred Scheel-Stiftung, Deutsche Krebshilfe e, V., Bonn, Germany. The authors wish to thank Mrs Berit Söhl, Mrs Birgit Kolon and Mrs Katja Dunckelmann for their expert technical assistance.

### References

COHEN, J.S., LYON, R.C., CHEN, C. & 5 others (1986). Differences in phosphate metabolite levels in drug-sensitive and -resistant human breast cancer cell lines determined by $^{31}P$ magnetic resonance spectroscopy. *Cancer Res.*, **46**, 4087.

COHEN, J.S. (1988). Phospholipid and energy metabolism of cancer cells monitored by $^{31}P$ MRS: possible clinical significance. *Mayo Clin. Proc.*, **63**, 1199.

CORBETT, T.H. & VALERIOTE, J.C. (1987). In *Rodent Tumour Models in Experimental Cancer Therapy* Kallman, R.F. (ed.), p. 233, Pergamon Press: N.Y.

COWAN, K.H., BATIST, G., TUKLPULE, A., SINHA, B.K. & MYERS, C.E. (1986). Similar biochemical changes associated with multidrug resistance in human breast cancer cells and carcinogen-induced resistance to xenobiotics in rats. *Proc. Natl Acad. Sci. USA*, **83**, 9228.

DALY, P.F., ZUGMAIER, G., SANDLER, D., CARPEN, M., MYERS, C.E. & COHEN, J.S. (1990). Regulation of the cytidine phospholipid pathways in human cancer cells and effects of 1- -D-arabinofuranosylcytosine: a noninvasive $^{31}P$ NMR study. *Cancer Res.*, **50**, 552.

EASTMAN, A. & SCHULTE, N. (1988). Enhanced DNA repair as a mechanism of resistance to cis-diammine-dichloroplatinum (II). *Biochemistry*, **27**, 4730.

EVELHOCH, J.L., SPARETO, S.A., NUSSBAUM, G.H. & ACKERMAN, J.J.H. (1986). Correlations between $^{31}P$ NMR spectroscopy and $^{15}O$ perfusion measurements in the RIF-1 murine tumor *in vivo*. *Rad. Res.*, **106**, 122.

EVELHOCH, J.L., KELLER, N.A. & CORBETT, T.H. (1987). Response-specific adriamycin sensitive markers provided by in vivo $^{31}P$ NMR spectroscopy in murine mammary adenocarcinomas. *Cancer Res.*, **47**, 3396.

GLICKSON, J.D. (1989). Clinical NMR spectroscopy of tumours. *Invest Radiol.*, **24**, 1011.

INAKA, M., KOBAYASHI, H., SAKURAI, Y. & JOHNSON, R.K. (1979). Active efflux of daunorubicin and adriamycin in sensitive and resistant sublines of P388 leukemia. *Biochem. Pharmacol.*, **27**, 2123.

JÄCKEL, M. & KÖPF-MAIER, P. (1990). Influence of cisplatin on cell cycle progression in xenographted human head and neck carcinomas. *Cancer Chemother. Pharmacol.* (submitted for publication).

LUTZ, N.W., LI, S.-J., WEHRLE, J.P. & GLICKSON, J.,D. (1988). *Phospholipid metabolites in chemically treated RIF-1 tumors monitored by in vivo $^{31}P$ NMR spectroscopy*. SMRM, sixth annual meeting, S.F. p. 398.

LYON, R.C., COHEN, J.S., FAUSTINO, P.J., MEGNIN, F. & MYERS, C.E. (1988). Glucose metabolism in drug-sensitive and drug-resistant human breast cancer cells monitored by magnetic resonance spectroscopy. *Cancer Res.*, **48**, 870.

MARIS, J.M., EVANS, A.E., McLAUGHLIN, A.C. & 4 others (1985). $^{31}P$ NMR spectroscopic investigation of human neuroblastoma *in situ*. *New Engl. J. Med.*, **312**, 1500.

MASUDA, H., OZOLS, R.F., LAI, G.-M., FOJO, A., ROTHENBERG, M. & MAMILTON, T.C. (1988). Increased DNA repair as a mechanism of acquired resistance to cisplatin in human ovarian cancer cell lines. *Cancer Res.*, **48**, 5713.

MÜLLER, W.E.G. (1975). *Chemotherapie von Tumoren – Biochemische Grundlagen*. Verlag Chemie: Weinheim.

POSNER, M.R., ERVIN, T., FABIAN, R.L. & 5 others (1984). The role of chemotherapy in treatment of advanced squamous cell cancer of the head and neck. *Laryngoscope*, **94**, 481.

SEKIYA, S., OOSAKI, T., ANDOH, S., SUZUKI, N., AKABOSHI, M. & TAKAMIZAWA, H. (1989). Mechanisms of resistance to cis-diamminedichloroplatinum (II) in a rat ovarian carcinoma cell line. *Eur. J. Cancer Clin. Oncol.*, **25**, 429.

SEO, K., MAURAKAMI, M., WATARI, H. & 4 others (1983). Intracellular pH determination by a $^{31}$P-NMR technique. The second dissociation constant of phosphoric acid in a biological system. *J. Biochem.*, **94**, 729.

SIJENS, P.E., DE JONG, W.H., SEIJKENS, D. & NEIJT, J.P. (1987). *$^{31}$P spectroscopy reveals an alkaline shift of pH in a cisplatin (CDDP) resistant tumor during treatment with CDDP*. SMRM, sixth annual meeting, S.F. p. 978.

SOSTMAN, D., ROCHWELL, S., SMITH, G.J.W. & 6 others (1988). MR, pathology and physiology of the BA1112 rhabdomyosarcoma *in vivo*. *Inv. Rad.*, **23**, 277.

STEEN, R.G. (1989). Response of solid tumors to chemotherapy monitored by *in vivo* $^{31}$P NMRS: a review. *Cancer Res.*, **49**, 4075.

SUNDQUIST, W.I. & LIPPARD, S.J. (1990). The coordination chemistry of platinum anticancer drugs and related compounds with DNA. *Coord. Chem. Rev.*, **100**, 293.

TOOHILL, R.J., ANDERSON, T., BYHARD, R.W. & 9 others (1987). Cisplatin and 5-fluorouracil as neoadjuvant therapy in head and neck cancer. *Arch. Otolaryngol Head Neck Surg.*, **113**, 758.

VAN DEN BOSCH, H. (1974). Phosphoglyceride metabolism. *Ann. Rev. Biochem.*, **43**, 243.

VAUPEL, P., OKUNIEFF, P., KALLINOWSKI, F. & NEURINGER, L.J. (1989). Correlations between $^{31}$P-NMR spectroscopy and tissue $O_2$ tension measurements in a murine fibrosarcoma. *Radiation Res.*, **120**, 477.